# Is Spousal Violence Being “Vertically Transmitted” through Victims? Findings from the Pakistan Demographic and Health Survey 2012-13

**DOI:** 10.1371/journal.pone.0129790

**Published:** 2015-06-17

**Authors:** Syeda Kanwal Aslam, Sidra Zaheer, Kashif Shafique

**Affiliations:** 1 School of Public Health, Dow University of Health Sciences, Karachi, Pakistan; 2 Institute of Health & Wellbeing, University of Glasgow, Glasgow, United Kingdom; Univ of Toledo, UNITED STATES

## Abstract

**Introduction:**

Violence against women is regarded as a major violation of human rights, and several socio-behavioral aspects among victims have been identified as important determinants of spousal violence experience. Pakistani nationally representative contextual evidence is scarce in this regard. We aimed to estimate prevalence of spousal violence, and explore its association with intergenerational transfer, and attitudinal acceptance of violence, among Pakistani ever-married women.

**Materials and Methods:**

Data of 3,687 ever-married women from Pakistan Demographic and Health Survey, 2012-13 was used to perform secondary analysis. Logistic regression analyses were conducted. Association between the different forms of spousal violence and the independent variables: intergenerational transfer of spousal violence (mother also beaten up by father); and attitudinal acceptance of spousal violence (beating is justifies if wife argues with husband) were reported as Odds ratios with 95% confidence intervals (CI).

**Results:**

Overall, more than a third (n=1344, 37.9%)of ever-married women reported that they experienced spousal violence. Almost 68% (n=539) of the women who reported that their mothers were also beaten up by their fathers, were victims of spousal violence; and almost 47% (n=603) of the women who agreed that beating was justified if the wife argues with her husband, also suffered spousal violence. Intergenerational transfer (OR =5.71, 95%CI 4.40-7.41, p-value <0.01), and attitudinal acceptance (OR =1.66, 95%CI 1.27-2.15, p-value <0.01) were significantly associated with experience of physical violence even after adjusting for respondents’ age at marriage, education level, wealth index, parity, employment status, and empowerment status.

**Conclusions:**

Spousal violence continues to haunt the lives of women in Pakistan, and is being transmitted as a learned behavior from mothers to daughters who tend to accept such violation of human rights. Girl children from such unfortunate homes may continue to transmit such behaviors, and thus may be targeted for future anti-domestic violence efforts.

## Introduction

Violence against women is regarded as a major violation of human rights. United Nations defines it as “any act of gender-based violence that results in, or is likely to result in, physical, sexual or mental harm or suffering to women, including threats of such acts, coercion or arbitrary deprivation of liberty, whether occurring in public or in private life"[1]. It may take several forms, amongst which spousal violence (SV) or intimate partner violence is a major perpetrator. It has been defined as “threatened, attempted, or completed physical, sexual or emotional abuse” committed by intimate partner in a relationship [2]. SV haunts the lives of millions of women worldwide, with almost 35% of women reporting the experience of SV globally [3]. It is associated with several adverse mental, and physical health outcomes among victims [4].

Various socio-demographic characteristics among victims have been identified as risk factors of SV, amongst which attitudinal acceptance or tolerant behavior towards SV among victims has been an important determinant [5]. Women may accept SV as part of their intimate relationship, and may give several justifications for SV acceptance related to cultural and religious norms like gender role transgressions, financial and emotional dependency, and self-blaming[6, 7]. They may choose to continue suffering at the hands of the perpetrator, and not comply with any anti-domestic violence effort at personal or social level to counteract such human right violation[8–10]. Such behavior at societal level, may aggravate the situation further, as it might favor the “acceptable climate for domestic violence” [9].

Further, children of unfortunate homes haunted by SV suffer immense social, mental, behavioral, and psychological problems[11]. The impact of SV may be greater on a girl child in a patriarchal society, where “acceptable climate for domestic violence” exists[9]. Women whose mothers also suffered SV at the hands of their fathers may accept SV more as part of the imbalanced power relationship experienced in a marriage [12]. Thus, mothers may “vertically transmit” a tolerant behavior towards SV to their daughters. Such behaviors may be transmitted over generations in societies where gender disparities are deeply rooted within cultures. Pakistani society may fit any such criterion well. The country ranks 126th among 149 countries, with Gender Inequality Index (GII) value of 0.563[13].

Very few studies have looked at Pakistani people’s attitude towards SV, and evidence regarding women’s attitude towards SV is limited[14]. Few studies have explored communities’ attitude towards SV. Fikree et al explored men’s attitudes towards wife beating, and found that majority of the perpetrators thought they had a right to beat their wives. Sheikh et al explored women’s views on religion’s standing on SV, and Anderson et al explored barriers to disclosing and reporting violence among women [15–17]. To the best of our knowledge none of the studies have explicitly explored these two inter-related behavioral aspects within Pakistani context. We thus aimed to estimate prevalence of SV among Pakistani ever married women, and explore association of SV with attitudinal acceptance of violence amongst women, and intergenerational transfer of violence.

## Materials and Methods

All details pertaining to the Pakistan Demographic and Health Survey (PDHS), 2012–13, are available online, and only the important aspects of methods are reported here[18].

### Study setting

Pakistan is a lower middle income country of Eastern Mediterranean region. The country has a predominant conservative, patriarchal, society; with severe gender related disparities. It currently ranks 126^th^ among 149 countries with Gender Inequality Index (GII) value of 0.563, and Gender Development Index (GDI) value of 0.750[13].

### Survey and Data

We used secondary data from nationally representative PDHS, 2012–13;conducted by the National Institute of Population Studies (NIPS) [18]. The data is owned by third party, and is available on The Measure DHS website. (URL: http://www.dhsprogram.com/data/available-datasets.cfm). This is the third survey from Pakistan, as part of the MEASURE DHS international series[18]. The survey aims to collect information related to demographic, maternal and child health indicators.

### Survey sampling frame

The sampling frame consisted of Pakistani urban and rural areas. Two stage stratified sample design was used. Overall, 500 primary sampling units (PSUs) were identified including urban and rural areas. At the second stage of sampling, 28 households were selected at each sampling point, through systematic random sampling. The estimated sample size for the survey was 14,000 households. 6944 households in urban areas and 7056 households in rural areas were selected. The survey was conducted in 498 areas, and 24 areas (mainly in Baluchistan province) were excluded due to adverse law and order situation.

### Eligibility criteria for women

One ever-married woman per household, aged between 15 and 45 years from every third household selected for PDHS 2012–13, were eligible to participate.

### Subsample for Domestic Violence (DV)

A total of 3743 women were eligible, out of which 3687were successfully interviewed. Forty three women were not interviewed because of failure to obtain privacy, and 13 due to some other reasons not reported in PDHS 2012–13 report. The participants were ever-married women, aged between 15 and 45 years. Overall survey response rate was 89.5%, and response rate for DV module was 98%.

### Ethical considerations

In order to account for the ethical considerations, WHO’s guidelines on ethical and safety recommendations for research on domestic violence were followed[19]. Random selection of one woman per household through Kish grid was used to ensure maintenance of confidentiality[20]. Besides initial consent for participation in PDHS survey, additional consent was taken before administering DV survey, and the participants were reassured regarding confidentiality. The DV survey was implemented on a condition of obtaining complete privacy. Although, WHO’s ethical and safety recommendations for research on domestic violence recommend provision of support material to all women surveyed for domestic violence; however, due to the sensitivity of the issue in Pakistani context, the researchers did not consider it suitable[19]. In case of identification of a DV victim, contact information of service centers was verbally provided by the interviewer.

### Data collection tool

The pretested, structured, questionnaires used for PDHS 2012–3 were developed to suit Pakistani cultural context of family planning, maternal and child health, DV, and HIV/AIDS related issues. The DV questionnaire used was shortened and modified version of the Conflict Tactics Scale (CTS)[21].

### Independent variables

The two main independent variables used in this study were:

1. Attitudinal acceptance of spousal violence (beating justified if wife argues with husband): In order to obtain information related to this aspect, following question was used: “In your opinion, is a husband justified in hitting or beating his wife, if she argues with him?” Response options were: Yes, No, and Do not know.
2. Intergenerational transfer of spousal violence (mother experienced spousal violence): In order to obtain information related to this aspect, following question was used: “As far as you know, did your father ever beat your mother?” Response options were: Yes, No, and Do not know.

In order to explore true effect of the attitudinal factors on SV, we used multivariate logistic regression analysis to adjust for any confounding variable [22]. Various important socio-demographic factors used in this study include:

Respondents’ age at marriage: In order to rule out effect of child marriage as confounder, this variable was selected[23].

Education level: Highest level of education obtained was noted. Response options include:

No education, Primary, Secondary (up to ten years of formal education), Higher (class 11 and above).

Wealth Index: Consisted of five categories: Poorest, poor, middle, richer, richest.

Employment status:

Empowerment status: Among several questions on empowerment related to household earnings, ownership of assets, and participation in household decisions, the question used as proxy for assessing woman’s empowerment status was: “Who is responsible for decisions regarding respondent’s health?” Women who responded that they themselves decided about their own health, and those who were involved in decision making along with their husband were labeled as “Wife involved in decision making”. All other responses (husband alone, family elders, other person) were labeled as “Wife not involved in decision making”.

### Dependent variables

All women were asked a series of questions to determine their experience of physical and emotional violence. All women who reported ever experiencing emotional or physical violence were noted. The survey questionnaire collects information about emotional violence first, and then moves to physical form of violence.

### Emotional violence

The questions used for recording information related to physical violence were:

Does (did) your (last) husband ever:

1. Say or do something to humiliate you in front of others?
2. Threaten to hurt or harm you or someone close to you?
3. Insult you or make you feel bad about yourself?

The response options were, “Yes”, and “No”. Women who answered “Yes” to any one of these questions were labeled as having “experienced emotional violence”. Women who answered “No” to all questions were labeled as “did not experience emotional violence”.

### Physical violence

The questions used for recording information related to physical violence were:

Does (did) your (last) husband ever:

1. Push you, shake you, or throw something at you?
2. Slap you?
3. Twist your arm or pull your hair?
4. Punch you with his fist or with something that could hurt you?
5. Kick you, drag you, or beat you up?
6. Try to choke you or burn you on purpose?
7. Threaten or attack you with a knife, gun, or any other weapon?

The response options were, “Yes”, and “No”. Women who answered “Yes” to any one of these questions were labeled as having “experienced physical violence”. Women who answered “No” to all questions were labeled as “did not experience physical violence”.

### Spousal violence

The third outcome variable, Spousal violence was developed by merging all forms of physical and emotional violence. Women who answered “Yes” to any of the above mentioned ten questions, were labeled as having “experienced spousal violence”, and those who answered “No” to all questions were labeled as “did not experience spousal violence”.

### Statistical analyses

We used SAS version 9.1.3 for data analysis. Complex survey data analysis was used, as PDHS follows multistage cluster sampling design. Primary sampling units, final weights, and strata were used to adjust for cluster sampling. Chi square test were used to determine significance of association between variables. [22, 23]. Univariate and multivariate logistic regression analyses were used, and Odds ratios with 95% confidence intervals were reported.

Firstly, association between the different forms of spousal violence and the independent variables: Intergenerational transfer of spousal violence; and attitudinal acceptance of spousal violence were reported in Model 0. Subsequently, Model 1 reports the results after adjusting for respondents’ age at marriage, education level, and wealth index. Thirdly, Model 2 reports the results after adjusting for respondents’ age at marriage, education level, wealth index, parity, employment status, and empowerment status.

## Results

The violence module of PDHS, 2013 was implemented among 3,743 ever-married women, of whom 3687 were successfully interviewed. The final analysis was conducted on information collected from 3545 women, after excluding 142 due to missing information on the variables selected for this study (empowerment status = 142, employment status = 38, beating justified if wife argues with husband = 23, spousal violence = 8, mother also experienced spousal violence = 4).

### Socio-demographic profile of respondents

Among respondents of the DV module, 42.2% (n = 1494) of the women were aged less than 18 years at time of marriage. 55.2% (n = 1959) were illiterate, and 37.9% (n = 1344) were poor i.e in lowest wealth quintile. Among them, 78.2% (n = 2771)were not employed in any waged work, and 47.2% (n = 1698) were not empowered to make decisions about their own health.

Overall, 1344(37.9%) of ever-married women reported that they experienced spousal violence (SV). Further, 794 (22.4%) of the women reported that their mothers also suffered from SV (inter-generational transfer of SV), and 1272(35.8%) women thought that beating was justified if wife argued with her husband (attitudinal acceptance of SV). Almost 68% (539) of the women who reported that their mothers experienced SV were victims of SV; and almost 47% (603) of the women who agreed that beating was justified if the wife argues with her husband, suffered SV.SV was significantly associated with inter-generational transfer of SV (p-value <0.01), and attitudinal acceptance of SV by the woman (p-value <0.01) (Table 1).

**Table 1:** Descriptive characteristics of women regarding experience of any form of spousal violence (physical/ emotional), PDHS 2012-2013(n = 3545).

Characteristics	Spousal Violence		
	Yes (n = 1344)	No (n = 2201)	
	n(%)	n(%)	p-value*
**Intergenerational transfer of spousal violence (mother also experienced spousal violence)**			
No	709 (28.5)	1781 (71.5)	< 0.01
Yes	539 (67.9)	255 (32.1)	
Do not know	96 (37.1)	165 (62.9)	
**Attitudinal acceptance of spousal violence (beating justified if wife argues with husband)**			
No	721 (32.3)	1513 (67.7)	< 0.01
Yes	603 (47.4)	669 (52.6)	
Do not know	20 (51.3)	19 (48.7)	
**Age at marriage**			
< 18	635 (42.5)	858 (57.5)	< 0.01
≥ 18	709 (34.6)	1343 (65.4)	
**Highest education level**			
No education	858 (43.8)	1100 (56.2)	< 0.01
Primary	199 (38.9)	313 (61.1)	
Secondary	183 (29.0)	448 (71.0)	
Higher	104 (23.4)	340 (76.6)	
**Wealth index**			
Poor	596 (44.4)	746 (55.6)	< 0.01
Middle	259 (39.8)	391 (60.2)	
Rich	489 (31.5)	1064 (68.5)	
**Parity**			
none	73 (21.6)	265 (78.4)	< 0.01
1–4	649 (34.6)	1226 (65.4)	
5+	622 (46.7)	710 (53.3)	
**Respondent's Employment status**			
Not working	1006 (36.3)	1765 (63.7)	< 0.01
Working	338 (43.7)	436 (56.3)	
**Respondent's empowerment status**			
Wife involved in decision-making	594 (35.0)	1105 (65.0)	< 0.01
Wife not involved in decision-making	750 (40.6)	1096 (59.4)	

* The p value has been calculated using Chi square test.

### Intergenerational transfer of SV (mother also experienced SV)

Univariate analysis (model 0) indicated that women who reported that their mothers also experienced spousal violence were more likely to experience physical violence from their spouses (OR = 6.25, 95%CI 4.97–7.86, p-value <0.01). (Table 2) The association remained significant after adjusting for respondent’s age at marriage, and wealth index in model 1(OR = 5.97, 95%CI 4.67–7.62, p-value <0.01). The association also remained significant in model 2, i.e. after adjusting for respondent’s age at marriage, wealth index, parity, employment status, and empowerment status (OR = 5.71, 95%CI 4.40–7.41, p-value <0.01).

**Table 2:** Factors associated with physical and emotional forms of spousal violence, PDHS 2012–2013 (n = 3545).

Characteristics	Model 0		Model 1		Model 2	
	OR (95% CI)	p-value	OR (95% CI)	p-value	OR (95% CI)	p-value
**Any form of spousal violence**						
**Intergenerational transfer of spousal violence (mother also experienced spousal violence)**						
No	1		1		1	
Yes	4.31 (3.40–5.45)	< 0.01	4.06 (3.16–5.22)	< 0.01	3.88 (3.00–5.02)	< 0.01
Do not know	1.21 (0.84–1.75)	0.29	1.11 (0.74–1.63)	0.61	1.07 (0.71–1.62)	0.73
**Attitudinal acceptance of spousal violence (beating justified if wife argues with husband)**						
No	1		1		1	
Yes	2.14 (1.77–2.58)	< 0.01	1.81 (1.48–2.21)	< 0.01	1.54 (1.24–1.90)	< 0.01
Do not know	2.66 (0.99–7.13)	0.05	2.50 (0.92–6.74)	0.06	2.77 (0.91–8.40)	0.07
**Physical violence**						
**Intergenerational transfer of spousal violence (mother experienced spousal violence)**						
No	1		1		1	
Yes	6.25 (4.97–7.86)	< 0.01	5.97 (4.67–7.62)	< 0.01	5.71 (4.40–7.41)	< 0.01
Do not know	1.70 (1.14–2.52)	< 0.01	1.55 (1.01–2.36)	0.04	1.55 (1.00–2.40)	0.04
**Attitudinal acceptance of spousal violence (beating justified if wife argues with husband)**						
No	1		1		1	
Yes	2.38 (1.90–2.99)	< 0.01	1.99 (1.56–2.54)	< 0.01	1.66 (1.27–2.15)	< 0.01
Do not know	0.65 (0.26–1.60)	0.35	0.60 (0.24–1.46)	0.26	0.53 (0.22–1.26)	0.15
**Emotional violence**						
**Intergenerational transfer of spousal violence (mother experienced spousal violence)**						
No	1		1		1	
Yes	3.22 (2.53–4.09)	< 0.01	3.01 (2.34–3.86)	< 0.01	2.86 (2.22–3.68)	< 0.01
Do not know	1.02 (0.67–1.53)	0.93	0.92 (0.59–1.43)	0.73	0.89 (0.55–1.41)	0.63
**Attitudinal acceptance of spousal violence (beating justified if wife argues with husband)**						
No	1		1		1	
Yes	1.92 (1.60–2.30)	< 0.01	1.63 (1.34–1.98)	< 0.01	1.43 (1.16–1.75)	< 0.01
Do not know	3.64 (1.52–8.74)	< 0.01	3.38 (1.41–8.08)	< 0.01	3.40 (1.17–9.85)	0.02

Model 0 = Univariate analysis

Model 1 = Model 0 + Age at marriage, respondent's education level, wealth index

Model 2 = Model 1 + parity, respondent's employment status, respondent's empowerment status

Similarly, univariate analysis (model 0) indicated that such women were also more likely to experience emotional violence from their spouses (OR = 3.22, 95%CI 2.53–4.09, p-value <0.01). The association remained significant in model 1(OR = 3.01, 95%CI 2.34–3.86, p-value <0.01), and also in model 2(OR = 2.86, 95%CI 2.22–3.68, p-value <0.01).

### Attitudinal acceptance of spousal violence (beating justified if wife argues with husband)

Women who agreed that beating is justified if wife argues with her husband were more likely to experience physical violence from their spouses in model 0 (OR = 2.38, 95%CI 1.90–2.99, p-value <0.01). The association remained significant in model 1(OR = 1.99, 95%CI 1.56–2.54, p-value <0.01), and also in model 2(OR = 1.66, 95%CI 1.27–2.15, p-value <0.01).

Similarly, univariate analysis (model 0) indicated that such women were also more likely to experience emotional violence from their spouses (OR = 1.92, 95%CI 1.60–2.30, p-value <0.01). The association remained significant in model 1(OR = 1.63, 95%CI 1.34–1.98, p-value <0.01), and also in model 2(OR = 1.43, 95%CI 1.16–1.75, p-value <0.01).

## Discussion

Our study results indicate that one in three of all Pakistani ever-married women suffer from spousal violence. Familial transfer of victimization and tolerant attitudes towards SV remain significant predictors of SV experience among women. The effect did not deter much even after adjusting for important covariates in the three models. It reflects the deep rooted acceptance of the abuse among victims. Pakistan ranks poorly among other countries on the gender development and inequality index, and women remain a major vulnerable population [13]. In a typical patriarchal society like Pakistan, where women in general, and wives in particular are expected to be submissive, culture dominantly controls issues like marital power, distribution of resources, and autonomy[24]. The victims may continue to suffer because they have been “encultured” that way in a society, where marital power lies with the husbands[8, 25].

We found a high prevalence of SV, consistent with other studies in the country [26, 27]. The prevalence is also comparable with global estimates as reported by WHO, according to which almost 35% of all women worldwide have experienced physical or emotional form of violence[22]. However, it may be important to note here that there are fair chances that SV is underreported in the given Pakistani religious and cultural context[28]. Researchers note that reported prevalence of domestic violence may be regarded as “the tip of the domestic violence iceberg”, where majority of the victims are still invisible to the society at large [9]. Unfortunately, it may be the underlying cause of several adverse health outcomes among the sufferers including physical, psychological, sexual, and reproductive issues, along with serious social and economic costs[29].

As far as socialization is concerned, parents and family serve as the primary socialization agent for a child, and it has been observed that children who observe family aggression during childhood exhibit an acceptability of family aggression as adults[30]. A child who’s witness to marital violence at home may suffer immensely from a range of behavioral and social problems, adversely affecting his/her quality of life[11]. The situation may be worse if the victim of such experiences is a girl child in a society with entrenched patriarchal system and gender related disparities[31]. Unfortunately, Pakistani women are victim of issues like high prevalence of spousal violence, and gender disparities; and such cultural norms have resulted in development of an overall tolerant attitude among females towards spousal violence[28, 32]. Our findings indicate such effect, and women who reported witnessing marital violence as a child were more likely to suffer from SV. Using regression analysis, we adjusted for the important correlates of SV, and intergenerational transfer of SV remained significantly associated with SV across models. Important social factors like age at marriage, wealth, parity, employment status and social empowerment, have not been able to deter the effect. In order to explain the association further, we also found that women were more likely to be victims of all types of SV if they were of the view that husbands are justified to hit wives in cases of arguments, or disobedience. The study builds on evidence that explored attitudinal acceptance for spousal violence at individual level [5, 33, 34].

Our study has various limitations that need to be considered while interpretation of results. Firstly, cross sectional nature of the study may limit the ability to draw causal inferences. We can’t rule out the speculation that women who experienced violence in their relationship have now adjusted their life accordingly, and have learned to cope with it by developing a tolerant behavior towards it. We need more extensive research, probably qualitative in nature, to explore the issue further. Secondly, the questionnaire assessed witnessing of SV as a child through a single question on mother’s experience of physical violence (“Did your father ever beat your mother?”), and in our study we have seen its association with both physical and emotional violence. There is a possibility of over/under estimation of the effect, nevertheless, the findings provides some initial clues, and give direction for further exploration. Thirdly, due to sensitivity of the issue in Pakistani conservative society, sexual form of spousal violence was not explored in PDHD 2012–13. The quantitative nature of the study and data collection may also result in missing out several important aspects associated with the spousal violence. Further, the validity of the PDHS questionnaire has not been compared with other population based surveys with similar social-demographic characteristics. Nevertheless, standardized DHS data collection procedures have been followed, and ensure credibility of results. Also, self-reported nature of the data, may demerit the internal validity of the study. Although the survey questionnaire uses 10 questions to collect information related to SV, given the victim blaming attitude of the society in our context, a woman might choose not to report her suffering, leading to intentional differential recall (reporting bias). Nevertheless, the survey probes an important sensitive issue of the society, and findings may hint towards the ground realities related to the issue. Lastly, PDHS 2012–13 records SV related information from the women only. It is important to note how these factors influence the perpetrators i.e. the husbands. We thus recommend that similar study must be conducted among men as well to explore vertical transmission of SV related factors among them. Nevertheless, the results of a nationally representative Pakistani study add to the limited evidence available in regard of SV in Pakistan. Proper sampling, sound methodology, nationwide coverage, are few of the many strengths of the demographic health survey. Further, SV was one of the core modules of the PDHS 2012–13, which focused on women and child health issues. Adequate training of field staff regarding data collection, confidentiality, and ethical considerations, add to the credibility of study results.

Spousal violence continues to haunt the lives of women in Pakistan, and is being transmitted as a learned behavior from mothers to daughters who tend to accept such abuse. They may continue to be harmed because they feel it a marital norm. We may not be able to reach out or help the victims; unless and until they at least realize that they are being subjected to basic human rights’ violation. No anti-domestic violence efforts may be effective in alleviating their problem, unless we are able to address the issue of their perceived ignorance of the matter. Anti-domestic violence policies need to focus on improving overall compliance of the victims with the anti-domestic violence efforts. Furthermore, specific behavior change interventions may target girl children from such unfortunate homes to halt intergenerational transfer of victimization among women. The findings may provide implications for public health efforts aiming at reducing women’s tolerance towards SV.

## References

[1] 1 Assembly UG. Declaration on the Elimination of Violence against Women. UN General Assembly. 1993.

[2] Control CfD, Prevention. Adverse health conditions and health risk behaviors associated with intimate partner violence—United States, 2005. MMWR: Morbidity and mortality weekly report. 2008;57(5):113–7. 18256582

[3] García-Moreno C, Jansen H, Ellsberg M, Heise L, Watts C. WHO multi-country study on women’s health and domestic violence against women. Geneva: World Health Organization 2005;204.

[4] Butchart A, García-Moreno C, Mikton C, Organization WH. Preventing intimate partner and sexual violence against women: taking action and generating evidence: World Health Organization; 2010. 10.1136/ip.2010.029629 20921563

[5] Uthman OA, Moradi T, Lawoko S. Are individual and community acceptance and witnessing of intimate partner violence related to its occurrence? Multilevel structural equation model. PloS one. 2011;6(12):e27738 10.1371/journal.pone.0027738 22194791 PMC3237419

[6] Jewkes R. Intimate partner violence: causes and prevention. The Lancet. 2002;359(9315):1423–9. 11978358 10.1016/S0140-6736(02)08357-5

[7] Wolf ME, Ly U, Hobart MA, Kernic MA. Barriers to seeking police help for intimate partner violence. Journal of Family Violence. 2003;18(2):121–9.

[8] Shrader E, Sagot M. Domestic violence: womens way out. 2000.

[9] Gracia E. Unreported cases of domestic violence against women: Towards an epidemiology of social silence, tolerance, and inhibition. Journal of epidemiology and community health. 2004;58(7):536–7. 15194711 10.1136/jech.2003.019604 PMC1732820

[10] Klein E, Campbell J, Soler E, Ghez M. Ending domestic violence: Changing public perceptions/halting the epidemic: Sage Publications, Inc; 1997.

[11] Kitzmann KM, Gaylord NK, Holt AR, Kenny ED. Child witnesses to domestic violence: a meta-analytic review. Journal of consulting and clinical psychology. 2003;71(2):339 12699028 10.1037/0022-006x.71.2.339

[12] Babcock JC, Waltz J, Jacobson NS, Gottman JM. Power and violence: the relation between communication patterns, power discrepancies, and domestic violence. Journal of consulting and clinical psychology. 1993;61(1):40 8450106 10.1037//0022-006x.61.1.40

[13] Malik K. Human Development Report 2014: Sustaining Human Progress: Reducing Vulnerabilities and Building Resilience. New York: United Nations Development Programme(http://hdrundporg/sites/default/files/hdr14-report-en-1 pdf). 2014.

[14] Ali PA, Naylor PB, Croot E, O’Cathain A. Intimate Partner Violence in Pakistan A Systematic Review Trauma, Violence, & Abuse. 2014:1524838014526065. 10.1177/1524838014526065 24626459

[15] Andersson N, Cockcroft A, Ansari U, Omer K, Ansari NM, Khan A, et al Barriers to disclosing and reporting violence among women in Pakistan: findings from a national household survey and focus group discussions. Journal of interpersonal violence. 2010;25(11):1965–85. 10.1177/0886260509354512 20007557

[16] Fikree FF, Razzak JA, Durocher J. Attitudes of Pakistani men to domestic violence: a study from Karachi, Pakistan. The journal of men's health & gender. 2005;2(1):49–58. 10.1161/CIRCOUTCOMES.114.001607 25714829

[17] Shaikh MA, Shaikh IA, Kamal A, Masood S. Domestic Violence and Pregnancy–Perspective from Islamabad and Rawalpindi. Journal of the College of Physicians and Surgeons Pakistan. 2008;18(10):662–3. 10.2008/JCPSP.662663 18940132

[18] NIPS II. Pakistan Demographic and Health Survey 2012–13. Isamabad2013. Available from: http://www.nips.org.pk/abstract_files/Priliminary%20Report%20Final.pdf.

[19] Garcia-Moreno C, Watts C, Heise L. Putting women first: Ethical and safety recommendations for research on domestic violence against women. Department of Gender and Women’s Health, World Health Organization Geneva, Switzerland 2001.

[20] Kish L. Survey sampling. 1965.

[21] Straus M, Hamby S, Boney-McCoy S, Sugarman D, Finkelhor D. Conflict tactics scales (CTS). 1973.

[22] WHO. Intimate partner and sexual violence against women 2014. Available from: http://www.who.int/mediacentre/factsheets/fs239/en/.

[23] Nasrullah M, Zakar R, Zakar MZ. Child marriage and its associations with controlling behaviors and spousal violence against adolescent and young women in Pakistan. Journal of Adolescent Health. 2014;55(6):804–9. 10.1016/j.jadohealth.2014.06.013 25123525

[24] Rabbani F, Qureshi F, Rizvi N. Perspectives on domestic violence: case study from Karachi, Pakistan. 2008. 18561735

[25] Yoshihama M, Blazevski J, Bybee D. Enculturation and attitudes toward intimate partner violence and gender roles in an Asian Indian population: Implications for community-based prevention. American journal of community psychology. 2014;53(3–4):249–60. 10.1007/s10464-014-9656-0 24515653

[26] Bibi S, Ashfaq S, Shaikh F, Qureshi PMA. Prevalenceinstigating factors and help seeking behavior of physical domestic violence among married women of HyderabadSindh. Pakistan journal of medical sciences. 2014;30(1):122 10.12669/pjms.301.4533 24639844 PMC3955555

[27] Fikree FF, Jafarey SN, Korejo R, Afshan A, Durocher JM. Intimate partner violence before and during pregnancy: experiences of postpartum women in Karachi, Pakistan. J Pak Med Assoc. 2006;56(6):252–7. 16827246

[28] Ali PA, Gavino MIB. Violence against women in Pakistan: a framework for Analysis. Journal of Pakistan Medical Association (JPMA). 2008. 18655430

[29] Valpied J, Hegarty K. Intimate partner abuse: identifying, caring for and helping women in healthcare settings. Women's Health. 2015;11(1):51–63. 10.2217/whe.14.59 25581055

[30] Kalmuss D. The intergenerational transmission of marital aggression. Journal of Marriage and the Family. 1984:11–9.

[31] Jejeebhoy SJ, Sathar ZA. Women's autonomy in India and Pakistan: the influence of religion and region. Population and development review. 2001;27(4):687–712.

[32] Pakeeza S. DOMESTIC VIOLENCE LAWS AND PRACTICES IN PAKISTAN. VFAST Transactions on Education and Social Sciences. 2015;6(1).

[33] Faramarzi M, Esmailzadeh S, Mosavi S. A comparison of abused and non-abused women's definitions of domestic violence and attitudes to acceptance of male dominance. European Journal of Obstetrics & Gynecology and Reproductive Biology. 2005;122(2):225–31. 15935543 10.1016/j.ejogrb.2004.11.047

[34] Gage AJ, Hutchinson PL. Power, control, and intimate partner sexual violence in Haiti. Archives of sexual behavior. 2006;35(1):11–24. 16502150 10.1007/s10508-006-8991-0

